# Supplementary material for: Noise-induced quantum synchronization with entangled oscillations
Source: Nat Commun. 2025 Sep 26;16:8457. doi: 10.1038/s41467-025-63196-6 (PMC12475214; doi:10.1038/s41467-025-63196-6)
Supplement: Supplementary file 1 — Supplementary Information [file 41467_2025_63196_MOESM1_ESM.pdf]

Supplementary Information: Noise-induced quantum synchronization with entangled oscillations

Ziyu Tao,[1, 2, 3, 4] Finn Schmolke,[5] Chang-Kang Hu,[1, 2, 3, 4] Wenhui Huang,[2, 3, 4] Yuxuan Zhou,[1, 2, 3, 4] Jiawei Zhang,[2, 3, 4] Ji Chu,[1, 2, 3, 4] Libo Zhang,[2, 3, 4] Xuandong Sun,[2, 3, 4] Zecheng Guo,[2, 3, 4] Jingjing Niu,[1] Wenle Weng,[6] Song Liu,[1, 2, 3, 4] Youpeng Zhong,[1, 2, 3, 4] Dian Tan,[1, 2, 3, 4] Dapeng Yu,[1, 2, 3, 4] and Eric Lutz[5]

[1]*International Quantum Academy, Futian District, Shenzhen, Guangdong 518048, China*
[2]*Shenzhen Institute for Quantum Science and Engineering and Department of Physics, Southern University of Science and Technology, Shenzhen 518055, China*
[3]*Guangdong Provincial Key Laboratory of Quantum Science and Engineering, Southern University of Science and Technology, Shenzhen 518055, China*
[4]*Shenzhen Key Laboratory of Quantum Science and Engineering, Southern University of Science and Technology, Shenzhen,518055, China*
[5]*Institute for Theoretical Physics I, University of Stuttgart, D-70550 Stuttgart, Germany*
[6]*Institute for Photonics and Advanced Sensing (IPAS), and School of Physics, Chemistry and Earth Sciences, The University of Adelaide, Adelaide, South Australia 5005, Australia*

Contents

I. Device characterization 2
II. Experimental setup 3
III. Gaussian noise generation 4
IV. Synchronization frequency for the five-qubit chain 5
V. Synchronized oscillations for longer spin chains 5
VI. Violation of the synchronization conditions 6
VII. Synchronization with a random initial state 9
VIII. Maximally entangled mixed states 10
IX. General formalism of quantum synchronization 12
X. Numerical simulations 13
References 13

I. Device characterization

In this experiment, we use two asymmetric Josephson junctions with $E_{J1}/E_{J2} = 3.0$ on the qubits, where E_{J1} and E_{J2} are the Josephson energies of the two junctions. The frequency (level spacing) ω_j of each qubit can be individually adjusted by varying the corresponding external flux through the Z control line and ranges from approximately 3.2 GHz to 4.6 GHz. In Fig. S1, we show the qubit frequencies of the linear chain of up to $N = 11$. In the main text, we focus on a consecutive set of five qubits as detailed below. In Fig. S2 we plot a typical relation between the tunable qubit frequency and the amplitude of applied flux.

Each qubit can be individually addressed and driven into the excited state by applying a microwave pulse through its XY control line. Figure S3 gives the energy relaxation (T_1) and dephasing time (T_2) of each qubit at its idling frequency. In the experiments involving 5, 8 and 11 working qubits, we choose these qubits from the index sets $\{4, 5, 6, 7, 8\}$, $\{4, 5, 6, \ldots, 11\}$ and $\{1, 2, 3, \ldots, 11\}$ respectively, where the frequency of the other unused qubits are largely detuned from the working qubits in the chosen index set of experiment, so the unused qubits do not influence

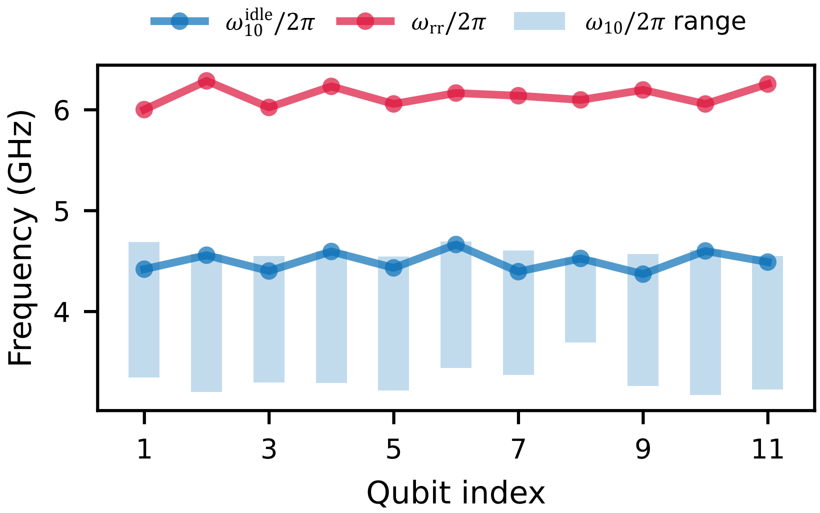

Supplementary Fig. S1. Individual qubit frequencies in our setup for a linear chain of up to $N = 11$ qubits. Blue dots denote the idle (unmodified) frequency $\omega_{10}/2\pi$ and the blue bars denote the bandwidth of each qubit. Red lines with dots denote the readout resonator frequencies $\omega_{rr}/2\pi$.

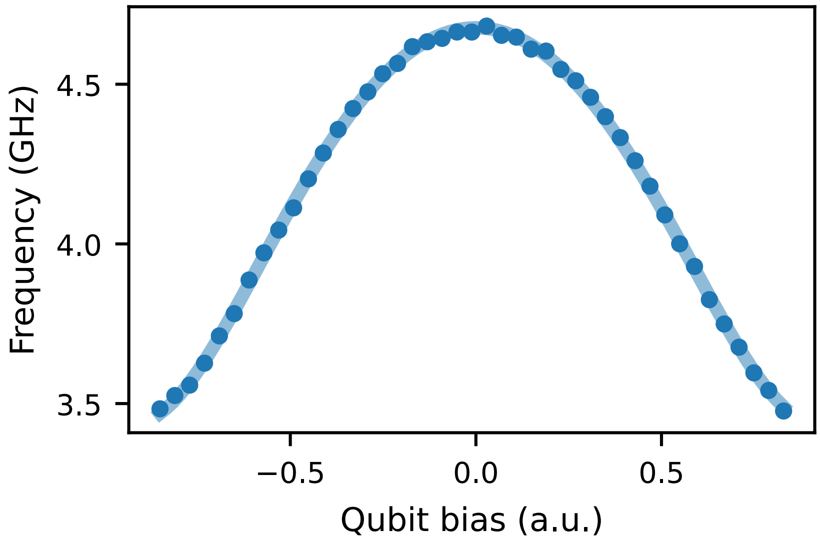

Supplementary Fig. S2. Typical relation between the qubit frequency and the flux amplitude (qubit bias) applied through Z control line.

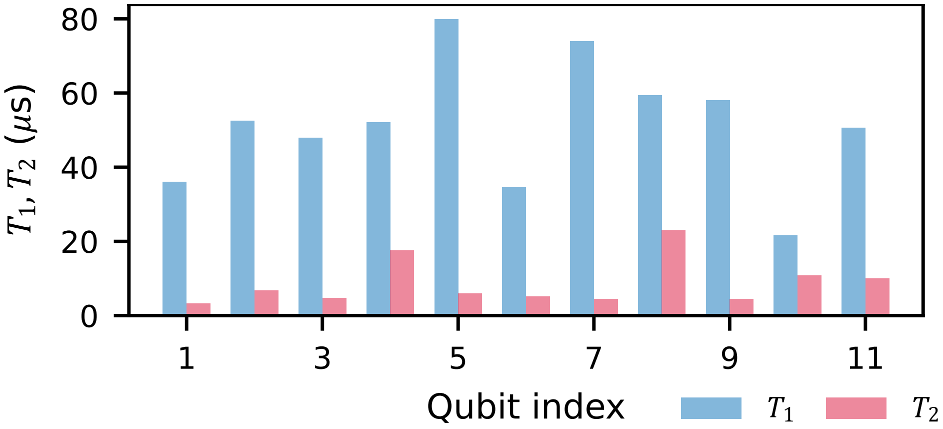

Supplementary Fig. S3. Qubit energy relaxation time T_1 and dephasing time T_2.

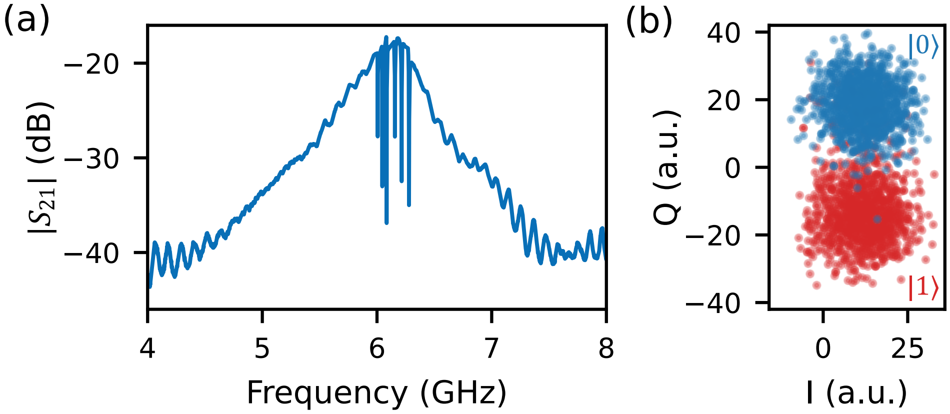

Supplementary Fig. S4. Qubit dispersive readout with Purcell filter. (a) Measured transmission spectrum of the Purcell filter. (b) Single-shot dispersive readout for states $|0\rangle, |1\rangle$ in the quadrature (IQ) space.

the desired evolution of the working qubits (5, 8 and 11 qubits in each experiment).

The dedicated readout resonator with frequency centered around 6.1 GHz is coupled to each qubit, where the state of the qubit can be deduced by measuring the state-dependent transmission of the readout resonator using the dispersive readout scheme. To mitigate qubit relaxation we use Purcell filters, where each group of 6 readout resonators is coupled to a Purcell filter with the center frequency of 6.1 GHz, which can be used as a bandpass filter to impede microwave propagation at the qubit frequency and suppress the Purcell decay rate, as seen in its transmission spectrum in Fig. S4(a). High-quality single-shot qubit dispersive readout for states $|0\rangle, |1\rangle$ can be achieved by using this Purcell filter, as shown in Fig. S4(b). Figure S5 displays the qubit readout fidelities, with an average state fidelity of 0.96 for the $|0\rangle$ state, 0.92 for the $|1\rangle$ state, respectively. A summary of the relevant experimental parameters for each qubit is provided in Table I.

The nearest-neighbour coupling strength between qubits can be controlled by applying external flux on the corresponding coupler [S1]. As shown in Fig. S6, we perform vacuum Rabi oscillation between the first excited states of $Q_{\rm A}$ and $Q_{\rm B}$ at different coupler bias to characterize the effective coupling strength between qubits, where the coupling strength $g_{\rm eff}$ can be continuously adjusted from +3.5 MHz to approximately −30 MHz [S2].

II. Experimental setup

Figure S7 shows the room temperature and cryogenic wiring layout used in our experiments, which is similar to our previous work [S3]. In the room temperature part of our setup, we use custom made digital-to-analog converter (DAC) and analog-to-digital converter (ADC) circuit boards for qubit control and readout, respectively. The control boards have dual-channel 14-bit vertical resolution DAC integrated circuits operating at 1 Gs/s driven by a field-

Qubit index	f_{10}^{idle} (GHz)	E_C (GHz)	E_{J1} (GHz)	E_{J2} (GHz)	f_{rr} (GHz)	F_0 (%)	F_1 (%)	T_1 (μs)	T_{2r} (μs)
1	4.420	0.239	9.71	2.99	6.002	94.9	93.6	36.10	3.3
2	4.559	0.215	10.05	3.26	6.287	93.3	91.4	52.60	6.8
3	4.402	0.221	9.94	2.93	6.024	98.2	92.7	47.90	4.7
4	4.595	0.212	10.45	3.20	6.232	95.3	93.6	52.10	17.6
5	4.433	0.221	9.78	3.08	6.058	96.3	92.2	79.90	6.0
6	4.665	0.216	10.85	3.10	6.166	94.3	86.4	34.60	5.2
7	4.396	0.221	10.25	2.93	6.140	96.8	94.6	74.00	4.4
8	4.527	0.216	10.95	2.09	6.098	97.1	91.9	59.40	22.9
9	4.371	0.224	9.80	3.02	6.197	97.0	94.7	58.10	4.6
10	4.601	0.213	10.19	3.46	6.058	94.3	89.7	21.60	10.8
11	4.491	0.222	9.77	3.05	6.255	96.8	94.8	50.70	10.1

Supplementary Table I. Device parameters. This table lists the parameters of 11-qubit 1D chain on a superconducting quantum processor including qubit frequencies, charging energy (E_C), Josephson energy (E_j), frequencies of readout cavities, readout fidelity and the coherence times for each qubit.

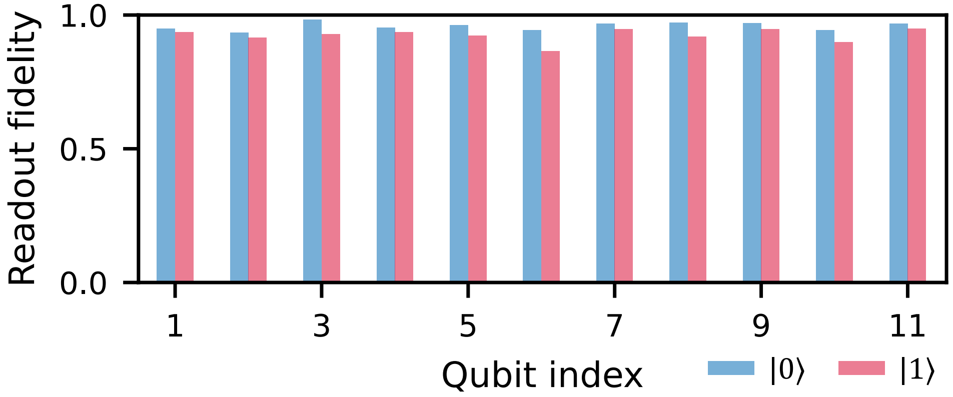

Supplementary Fig. S5. Qubit readout fidelities for states $|0\rangle$ and $|1\rangle$.

programmable gate array (FPGA) chip, where each DAC analog output is filtered by a custom Gaussian low-pass filter with 250 MHz bandwidth to filter the clock feedthrough. The DAC boards can generate nanosecond-length pulses for fast qubit Z or coupler C control, or modulate the in-phase and quadrature components of an IQ mixer for frequency up-conversion by using two channels, which provides several GHz frequency signals for qubit XY control and dispersive readout. In the cryogenic wiring, two cryogenic circulators are inserted between the qubits chip and the cryogenic HEMT to isolate reflections as well as thermal noise emitted from the input of the cryogenic HEMT to the chip, each control line is heavily attenuated and filtered at each temperature stage in the dilution refrigerator to minimize the impact on the qubit coherence while retaining controllability.

III. Gaussian noise generation

To inject the artificial noise into a superconducting qubit, we use the method discussed in Ref. [S4]. We apply a series of pulse sequences in the experiments and treat the outputs as an ensemble of trajectories, whose average gives an effective open quantum system evolution for the qubits. Each pulse sequence consists of successive 4-ns-wide square pulses, which are specifically designed to yield the desired noise spectrum. The noise spectral density $S(\omega)$ is first discretized into the series $S(\omega_m)$, where $\omega_m = 2\pi m/(Nk\tau_0)$ $(m = 0, 1, 2, \cdots, Nk/2)$, $\tau_0 = 4$ ns, N is the number of pulse sequences, k is the maximum number of successive 4-ns-wide pulses in each sequence, and $k\tau_0$ is the time duration of each sequence. For Gaussian white noise, $S(\omega)$ is simply a constant. We then multiply $\sqrt{S(\omega_m)}$ by a random phase factor θ_m which is randomly chosen from 0 to 2π, and perform the inverse Fourier transform of the

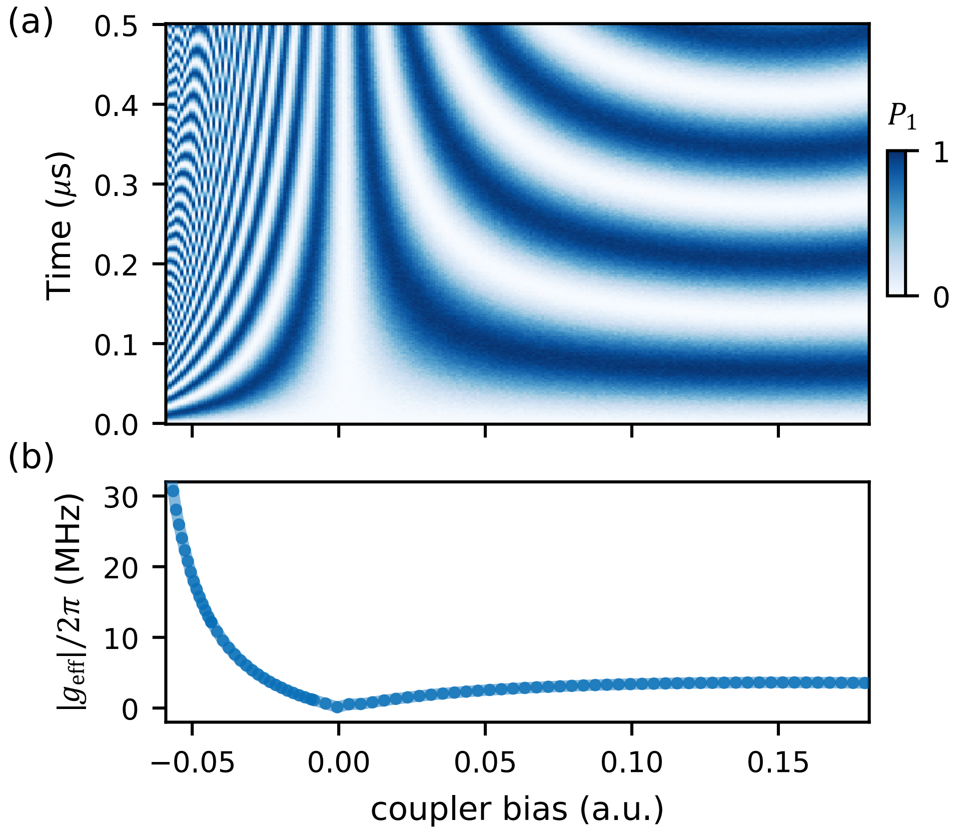

Supplementary Fig. S6. Characterization of tunable coupling. (a) Vacuum Rabi oscillation between $Q_{\rm A}$ and $Q_{\rm B}$ at different coupler bias, where the colors denote the population P_1 of $Q_{\rm B}$. (b) The tunable coupling strength $g_{\rm eff}$ extracted from the data of (a).

frequency series $\{\sqrt{S(\omega_m)}\exp(i\theta_m)\ , m = 0, 1, \cdots Nk/2\}$ to generate a discrete white noise time series $\xi(t)$ consisting of Nk numbers. The time series $\xi(t)$ is sliced into N sections providing the individual noise trajectories, which are transformed to the amplitudes of square pulses as experimentally generated by the digital-to-analog converter (arbitrary waveform generator). Figure S8 illustrates a pulse sequence of white Gaussian noise.

IV. Synchronization frequency for the five-qubit chain

The synchronization frequency $2J$ is determined by the coupling constant of the spin chain and not by the eigen-frequencies of the individual qubits. Figure S9 shows a fit the magnetizations of qubits $2-4$ for the five-qubit chain of the main text with the function $c_1\cos(2Jt) + c_2$.

V. Synchronized oscillations for longer spin chains

In this section, we demonstrate the synchronized magnetization $\langle\sigma_j^z\rangle$ in a chain of length $N = 8$ and $N = 11$. For $N = 8$, Gaussian white noises are locally applied on the third and sixth site $u = 3, 6$ with the reduced noise amplitude $\gamma \approx 0.5$. Figure S10 gives the measured magnetization $\langle\sigma_j^z\rangle$ in a 8-qubit chain, for the initial state with a single excitation $|\Psi(t = 0)\rangle = |1\rangle_1$ and two excitations $|\Psi(t = 0)\rangle = |1\rangle_1 \otimes |1\rangle_5$, as shown in Fig. S10(a) and Fig. S10(b). The evolution of $\langle\sigma_j^z\rangle$ shows a synchronous behavior for $\langle\sigma_{1,5,7}^z\rangle$ and $\langle\sigma_{2,4,8}^z\rangle$. Figure S11 shows the Pearson correlation coefficients extracted from Fig. S10. For $N = 11$, Gaussian white noises are locally applied on the sites $u = 3, 6, 9$ with the reduced noise amplitude $\gamma \approx 0.3$. Figure S12 gives the measured magnetization $\langle\sigma_j^z\rangle$ in a 11-qubit chain, for the initial state with single excitation $|\Psi(t = 0)\rangle = |1\rangle_1$, while Fig. S13 displays the corresponding Pearson correlation coefficient. Synchronization becomes more challenging for larger chains due to dissipation, nonuniform couplings and excitation of higher energy levels. In all cases, all eigenmodes are quickly suppressed until only a single one survives

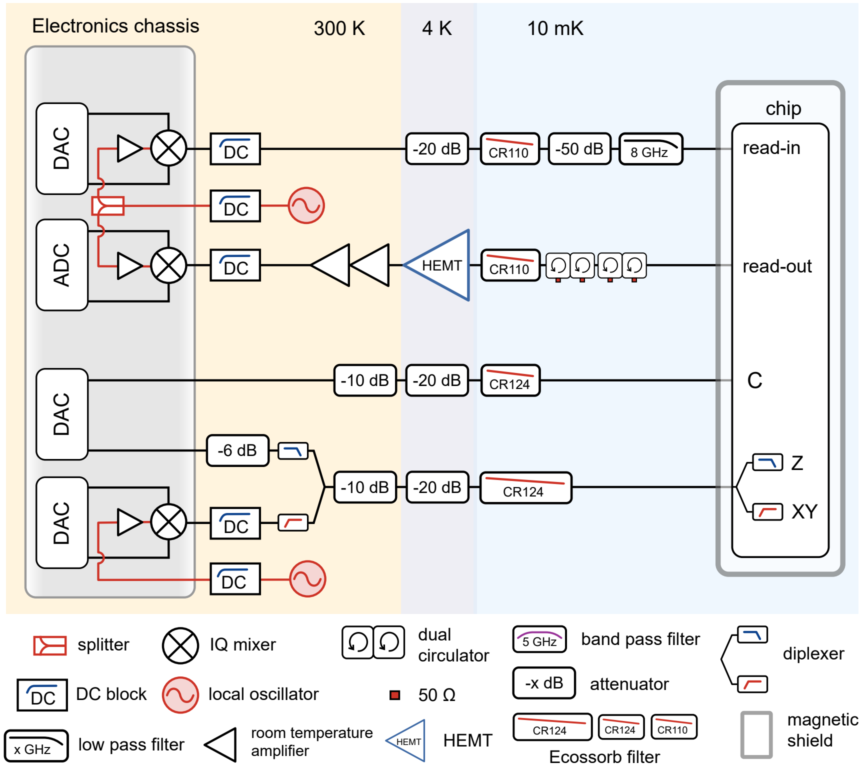

Supplementary Fig. S7. Room temperature and cryogenic wiring for the qubit control and readout.

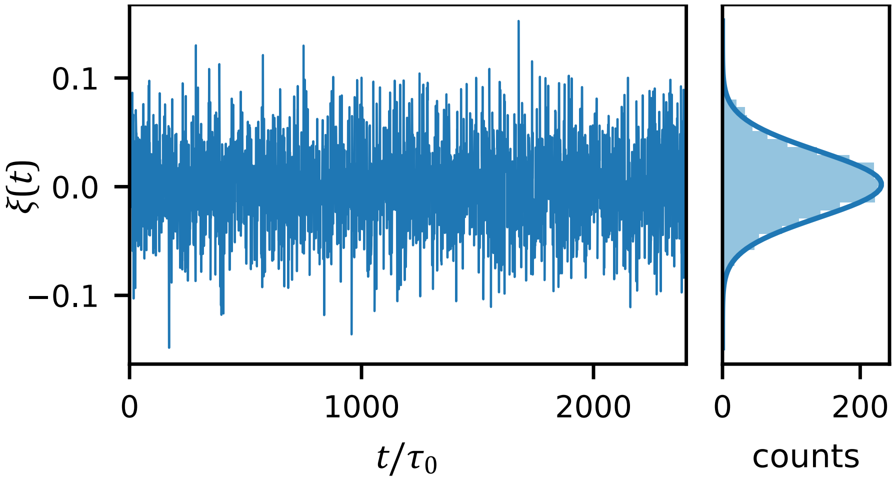

Supplementary Fig. S8. Illustration of the pulse sequence injecting the white noise with time series $\xi(t)$. The amplitude of white noise obeys the Gaussian distribution.

giving rise to synchronized oscillations across the entire system at a single frequency.

VI. Violation of the synchronization conditions

Synchronization with a single frequency is only possible in the XY chain with noise if the synchronization conditions is satisfied (see main text). When the conditions for transient synchronization are not obeyed, the quantum system will exhibit damped oscillations resulting in a time-independent steady state (no oscillations), cf. Ref. [S5]. In this section, we experimentally demonstrate this situation with the five-qubit example of the main text. Figure S14

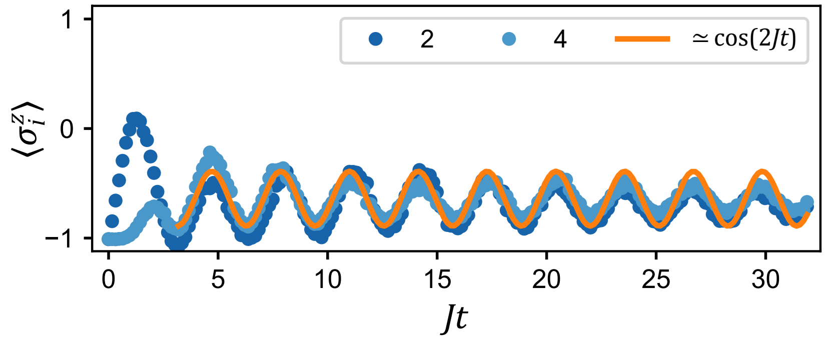

Supplementary Fig. S9. The synchronized magnetization $\langle \sigma_j^z \rangle$ oscillates with the period of $2J$, which fits a cosine-like function $c_1 \cos(2Jt) + c_2$ with constants c_1, c_2.

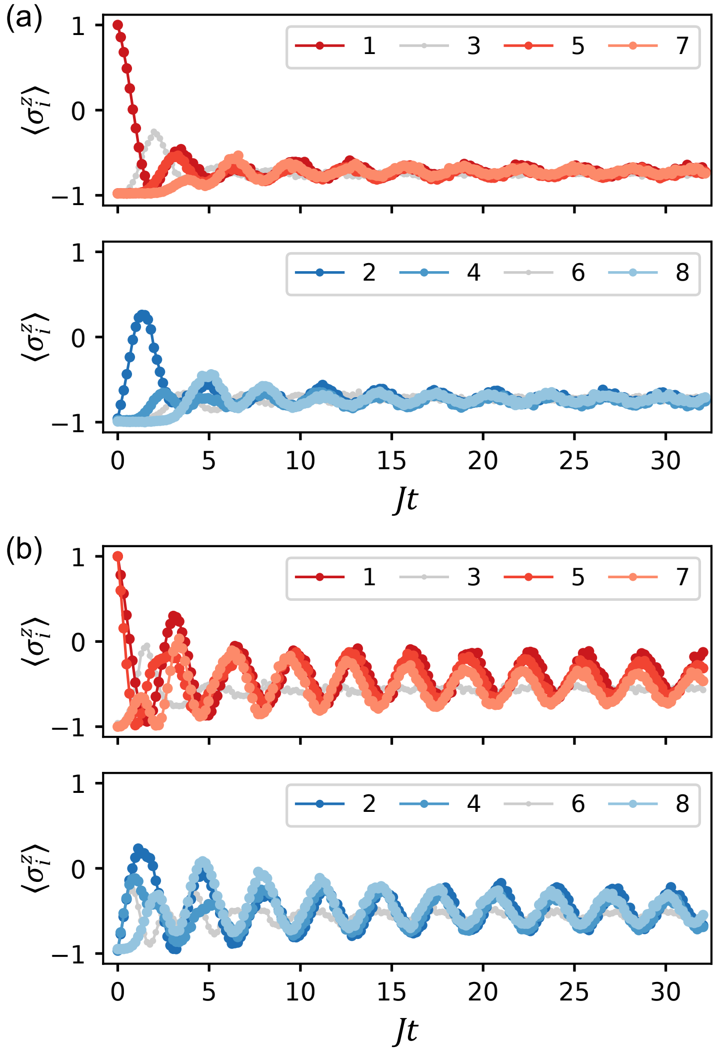

Supplementary Fig. S10. (a)-(b) Experimental demonstration of noise-induced synchronization in a XY model with $N = 8$ qubits. Measured magnetization $\langle \sigma_j^z \rangle$ in the 8-qubit chain. The system is initially prepared in the state (a) $|\Psi(t=0)\rangle = |1\rangle_1$, (b) $|\Psi(t=0)\rangle = |1\rangle_1 \otimes |1\rangle_5$.

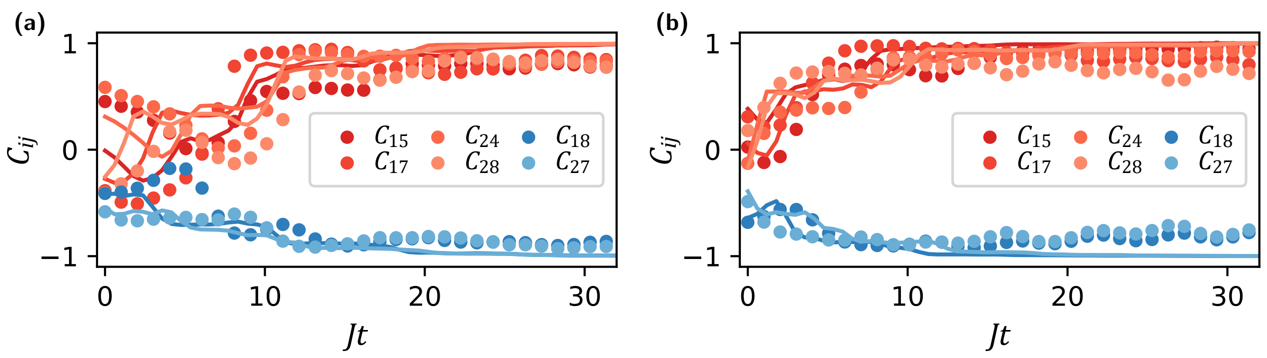

Supplementary Fig. S11. Pearson correlation coefficients extracted from the evolution of $\langle\sigma_j^z\rangle$ in a 8-qubit chain (dots) and numerical simulation (solid line) with the initial state (a) $|\Psi(t=0)\rangle = |1\rangle_1$, (b) $|\Psi(t=0)\rangle = |1\rangle_1 \otimes |1\rangle_5$.

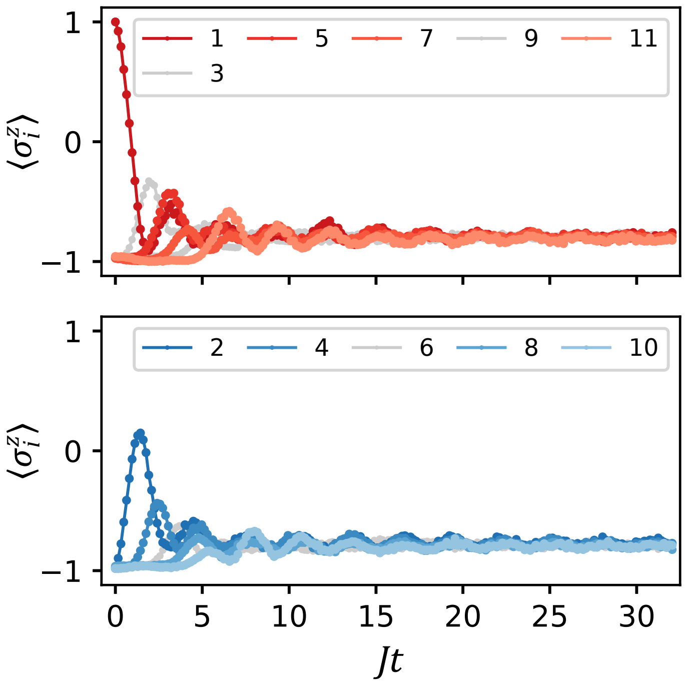

Supplementary Fig. S12. Experimental demonstration of noise-induced synchronization in a XY model with $N = 11$ qubits. Measured magnetization $\langle\sigma_j^z\rangle$ in a 11-qubit chain with the initial state $|\Psi(t=0)\rangle = |1\rangle_1$.

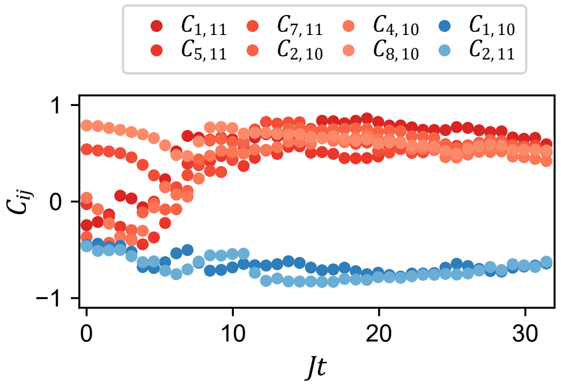

Supplementary Fig. S13. Pearson correlation coefficients of the $N = 11$ qubit chain for the single excitation initial state shown in Fig. S12. Synchronization becomes more challenging for larger chains due to dissipation, nonuniform couplings and excitation of higher energy levels.

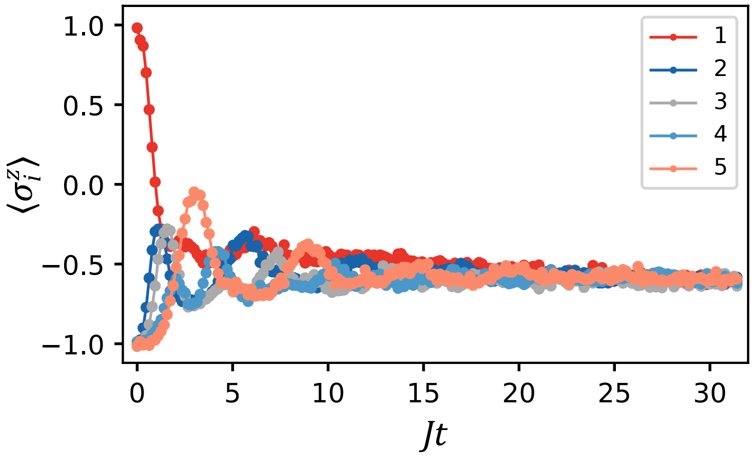

Supplementary Fig. S14. Evolution of the five-qubit quantum XY chain with Gaussian white noise applied to the first site $V = \sigma_1^z$. The synchronization condition is not satisfied and the qubits reach a time-independent stationary state without oscillations (same parameters as in the main text).

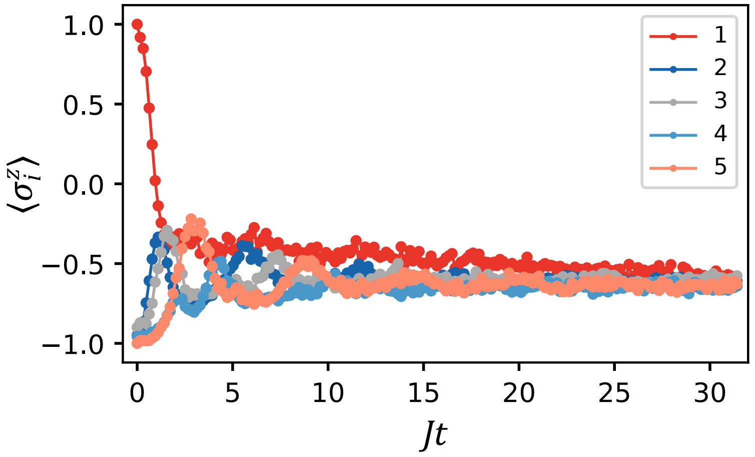

Supplementary Fig. S15. Evolution of the five-qubit quantum XY chain with Gaussian white noise applied to the second site $V = \sigma_2^z$. The synchronization condition is not satisfied and the qubits reach a time-independent stationary state without oscillations (same parameters as in the main text).

(Fig. S15) show the five-qubit evolution where Gaussian white noise is applied to the first site $V = \sigma_1^z$ (second site $V = \sigma_2^z$). As predicted by theory, we find no synchronization in this case.

VII. Synchronization with a random initial state

Noise-induced synchronization appears for a wide range of initial states. This is because the decoherence-free subspace responsible for the occurrence of synchronization belongs to the unique stable invariant manifold of the evolution, and thus attracts all trajectories in Hilbert space. This means that regardless of how the qubits are initialized, eventually there will be synchronization as long as the initial state has finite overlap with the synchronized mode. To show this, we consider the solution to the Lindblad equation (S20) in Liouville-space [S6]

$$|\rho(t)\rangle\!\rangle = \exp(\mathcal{L})\,|\rho(0)\rangle\!\rangle\,. \tag{S1}$$

Since $\mathcal{L}$ is not generally a normal operator, $[\mathcal{L}, \mathcal{L}^\dagger] \neq 0$, it cannot be unitarily diagonalized and, in particular, the left eigenvectors $\langle\!\langle\beta_k|$ do not coincide with the right eigenvectors $|\alpha_k\rangle\!\rangle$. The spectral decomposition thus yields

$$|\rho(t)\rangle\!\rangle = \sum_k e^{\alpha_k t}\,\langle\!\langle\beta_k|\rho(0)\rangle\!\rangle\,|\alpha_k\rangle\!\rangle\,. \tag{S2}$$

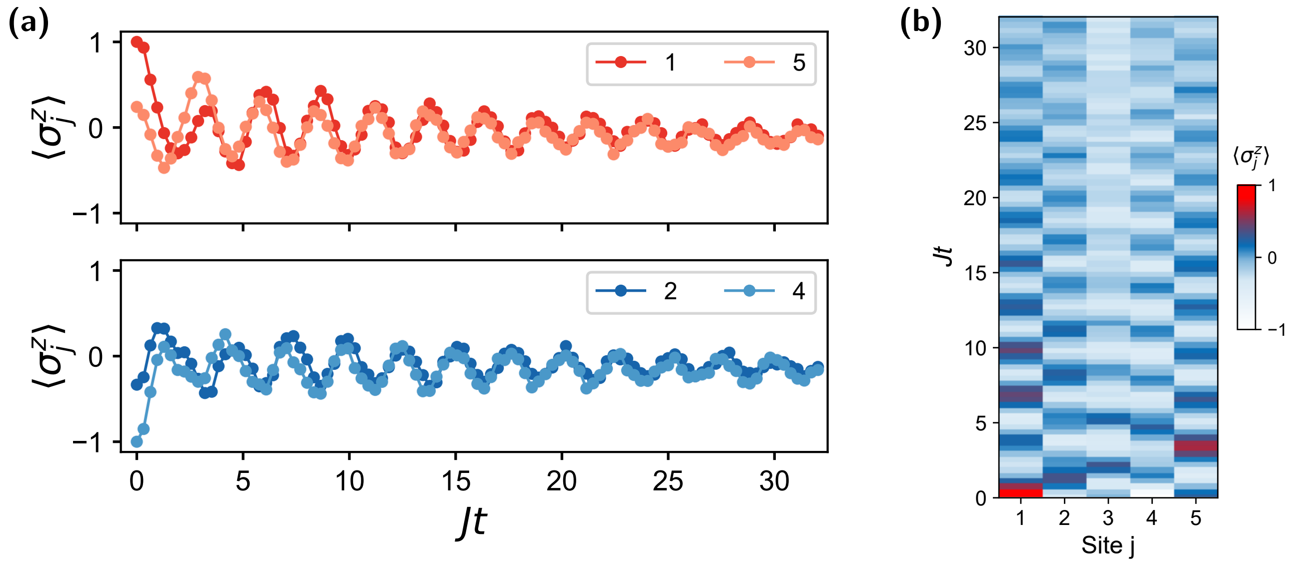

Supplementary Fig. S16. Noise-induced synchronization for a randomly selected initial state in (a) conventional representation and (b) in a stroposcopic map representation. Both (a) and (b) clearly demonstrate the onset of synchronization after a time $Jt \approx 15$. The initial state is the state $\rho(0) = \bigotimes_{j=1}^{5} (p_j |1\rangle\langle 1| + (1-p_j)|0\rangle\langle 0|)$, with $p_1 = 1, p_2 = 0.3, p_3 = 1/2, p_4 = 0, p_5 = 0.6$.

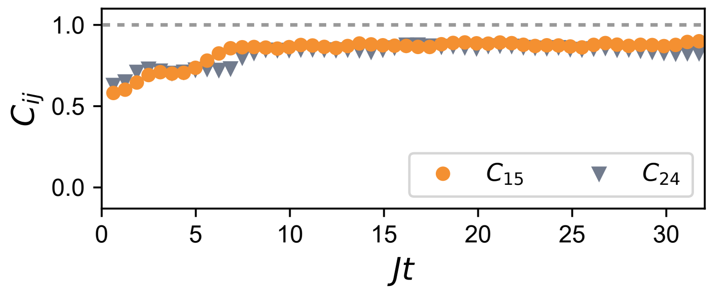

Supplementary Fig. S17. Pearson correlation coefficients of the $N = 5$ qubit chain for the random initial state shown in Fig. S16.

The eigenvalues may be decomposed into real and imaginary part, $\alpha_k = \mu_k + i\lambda_k$, where the real part $\mu_k < 0$ leads to exponential decay of the kth mode. In the long time limit, the dynamics will then be dominated by the coherent oscillations in the decoherence-free subspace for which $\alpha_k = i\lambda_k$ is purely imaginary

$$\lim_{t\to\infty} |\rho(t)\rangle\rangle = \sum_{\alpha_k,\beta_k \notin \mathrm{DFS}} \langle\langle \beta_k|\rho(0)\rangle\rangle\, |\alpha_k\rangle\rangle + \sum_{j,k} e^{-i(\lambda_j-\lambda_k)t} \langle\langle \nu_j,\nu_k|\rho(0)\rangle\rangle\, |\nu_j,\nu_k\rangle\rangle\,. \tag{S3}$$

Thus, to observe stable oscillations in the long time dynamics, the overlap should be nonzero, $\langle\langle \nu_j,\nu_k|\rho(0)\rangle\rangle \neq 0$ for $j \neq k$.

In the main text, we have demonstrated quantum correlated coherent oscillations for the pure, separable state $|\Psi\rangle = |1\rangle \otimes |0\rangle^{\otimes(N-1)}$ which is easy to prepare experimentally. However, as explained above, a wide range of initial configurations might be chosen. In Fig. S16 we further show in an additional experiment that an arbitrarily chosen mixed state also gives rise to noise-induced synchronization.

VIII. Maximally entangled mixed states

The quantum XY chain subject to noise on the third site possess a decoherence-free subspace that supports a single frequency, the synchronization frequency $\hbar\Lambda_{kl} = 2J$ [S5]. In this section, we theoretically show that, as long as the synchronization conditions are satisfied (see main text), the two states that give rise to the decoherence-free eigenmode, lead to maximally entangled mixed states between the end-spins of the chain for arbitrary system size N.

To simplify the calculations, we can either perform a Jordan-Wigner transformation [S5] or only consider the subspace of a single excitation (since, even in the presence of noise, the total magnetization is conserved). In both cases, we obtain a von Neumann equation with an effective tridiagonal Hamiltonian [S5]

$$\Omega/\hbar = \mathrm{diag}(J; 2\omega; J). \tag{S4}$$

The eigenstates of this matrix are given by [S7]

$$|\varphi_k\rangle = \left(\sin\left(\frac{\pi k}{N+1}\right), \sin\left(\frac{2\pi k}{N+1}\right), \ldots, \sin\left(\frac{N\pi k}{N+1}\right)\right)^{\mathrm{T}} \sqrt{\frac{2}{N+1}}, \tag{S5}$$

The two decoherence-free states that give rise to non-decaying oscillations in the long time limit have already been identified in Ref. [S5] and are given by $k = (N+1)/3$ and $l = 2(N+1)/3$, yielding

$$|\varphi_k^{\mathrm{s}}\rangle = \left(\sin\left(\frac{\pi}{3}\right), \sin\left(\frac{2\pi}{3}\right), \ldots, \sin\left(\frac{N\pi}{3}\right)\right)^{\mathrm{T}} \sqrt{\frac{2}{N+1}}, \tag{S6}$$

$$|\varphi_l^{\mathrm{s}}\rangle = \left(\sin\left(\frac{2\pi}{3}\right), \sin\left(\frac{4\pi}{3}\right), \ldots, \sin\left(\frac{2N\pi}{3}\right)\right)^{\mathrm{T}} \sqrt{\frac{2}{N+1}}. \tag{S7}$$

Transforming back into original Hilbert space, these states become

$$\begin{aligned} |\nu_1\rangle &= \sqrt{\frac{2}{N+1}} \sum_{n=1}^{N} \sin\left(\frac{n\pi}{3}\right) |1\rangle_n, \\ |\nu_1\rangle &= \sqrt{\frac{2}{N+1}} \sum_{n=1}^{N} \sin\left(\frac{2n\pi}{3}\right) |1\rangle_n, \end{aligned} \tag{S8}$$

where $|1\rangle_n = |0\rangle^{\otimes(n-1)} \otimes |1\rangle \otimes |0\rangle^{\otimes(N-n)}$ is the single excitation with excitation at site n. Upon performing the partial trace over all subsystems except the edge qubits, we arrive at

$$\rho_{1,N} = \mathrm{tr}_{[2,N-1]}(|\nu_1\rangle\langle\nu_1|) = \frac{2}{N+1} \sum_{m,n,k} \sin\left(\frac{n\pi}{3}\right) \sin\left(\frac{m\pi}{3}\right) \mathrm{tr}_{[2,N-1]}(|1\rangle_n \langle 1|_m), \tag{S9}$$

$$= \frac{2}{N+1} \left[\frac{2(N-2)}{3} \sin\left(\frac{\pi}{3}\right)^2 |00\rangle\langle 00| + \sin\left(\frac{N\pi}{3}\right)^2 |01\rangle\langle 01| \right. \tag{S10}$$

$$+ \sin\left(\frac{(N-1)\pi}{3}\right)^2 |10\rangle\langle 10| \tag{S11}$$

$$\left. + \sin\left(\frac{N\pi}{3}\right) \sin\left(\frac{(N-1)\pi}{3}\right) (|01\rangle\langle 10| + |10\rangle\langle 01|) \right]. \tag{S12}$$

Since, $\sin(n\pi/3)^2 = 3/4$ for $n \in \mathbb{N}$, both decoherence-free states share the same reduced two-qubit state, $\rho_{1,N} = \mathrm{tr}_{[2,N-1]}(|\nu_1\rangle\langle\nu_1|) = \mathrm{tr}_{[2,N-1]}(|\nu_2\rangle\langle\nu_2|)$. This two-qubit mixed state can be conveniently expressed in matrix form

$$\rho_{1,N} = \frac{3}{2N+2} \begin{pmatrix} 0 & 0 & 0 & 0 \\ 0 & 1 & -1 & 0 \\ 0 & -1 & 1 & 0 \\ 0 & 0 & 0 & \frac{2N-4}{3} \end{pmatrix} = M. \tag{S13}$$

States of this form belong to the class of maximally entangled mixed states and can be parametrized as [S8]

$$M = p_1 \left|\Psi^-\right\rangle\left\langle\Psi^-\right| + p_2 |00\rangle\langle 00| + p_3 \left|\Psi^+\right\rangle\left\langle\Psi^+\right| + p_4 |11\rangle\langle 11|, \tag{S14}$$

where $\sum_k p_k = 1$ and, crucially, the hierarchy $p_1 \leq p_2 \leq p_3 \leq p_4$ has to be respected. Here, the states $|\Psi^-\rangle = (|01\rangle - |10\rangle)/\sqrt{2}$ and $|\Psi^+\rangle = (|01\rangle + |10\rangle)/\sqrt{2}$ are the usual Bell states. For the above states, we find

$$p_1 = \frac{3}{N+1}, \quad p_2 = \frac{N-2}{N+1}, \quad p_3 = p_4 = 0. \tag{S15}$$

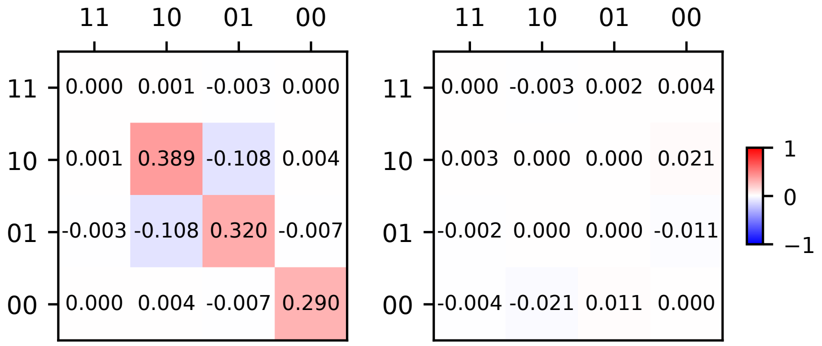

Supplementary Fig. S18. The real (left) and imaginary (right) parts of the experimentally extracted density matrix ρ_{15} of the two end qubits in the synchronized regime of 5-qubit experiment at $Jt = 3\pi$.

The purity, $\mathrm{tr}\left(\rho_{1,N}^2\right) = (9 + (N-2)^2)/(N+1)^2$, and the concurrence, $C(\rho_{1,N}^2) = 3/(N+1)$, immediately follow [S8]. The concurrence is nonzero for all finite N.

Since, $|\nu_{1,2}\rangle$ are decoherence-free subspaces, they are in the kernel of the Liouvillian, $\mathcal{L}\,|\nu_{1,2}\rangle\langle\nu_{1,2}| = 0$, and thus belong to the invariant states if they are excited by the initial condition. In the main text, the initial condition for noise-induced synchronization is $|\Psi(0)\rangle = |1\rangle_1$. Both states have a non-zero overlap of $|\langle\nu_{1,2}|\Psi(0)\rangle|^2 = 3/(2N+2)$ with the initial state. Consequently, the stationary state contains the maximally entangled mixed state contribution $3/(2N+2)(|\nu_1\rangle\langle\nu_1| + |\nu_2\rangle\langle\nu_2|)$.

Real and imaginary parts of the experimentally reconstructed state ρ_{15} at $Jt = 3\pi$ (corresponding to Fig. 1f of the main text) are presented in Fig. S18. The real part has the form of a maximally entangled mixed state (the fidelity with Eq. (S13) is 99.3%), while the imaginary part is basically zero.

IX. General formalism of quantum synchronization

In this section, we relate the phenomenon of noise-induced quantum synchronization to the general formalism of Ref. [S9] which provides necessary and sufficient conditions for local observables to be identically synchronized. To obtain undamped oscillations, purely imaginary eigenvalues of the Lindbladian $\mathcal{L}$ are required. This condition is of course trivially satisfied by closed quantum systems under unitary evolution, however, for arbitrary initial states, synchronization between local observables is generally not expected, since the resulting superposition of eigenmodes will not be permutation-invariant with respect to different sites. Dissipative dynamics is therefore necessary to single out such permutation invariant modes, and thereby establish synchronized oscillations in the long time dynamics.

Existence of purely imaginary eigenvalues requires steady-state degeneracy of the Lindbladian, which is guaranteed if there exists a strong dynamical symmetry operator A [S10–S12]. A strong dynamical symmetry is a conserved quantity of the Liouvillian, $\mathcal{L}^\dagger(A) = 0$, that satisfies $[H, A] = \omega A$ and $[A, L] = [A, L^\dagger] = 0$. Finding a suitable A is usually not straightforward because it might correspond to an abstract hidden symmetry of the Hilbert space that does not admit an obvious physical interpretation. To find the operator A^{sync} responsible for noise-induced synchronization, we have performed perturbation theory in Liouville space, from which we could obtain the synchronized eigenstates, the corresponding eigenmode and the synchronized frequency exactly [S5]. Their explicit form is given in Eq. (S8). From Eq. (S3) we can compute the synchronized eigenmode, which is given by [S5]

$$|\epsilon\rangle^{\mathrm{s}} = \begin{bmatrix} \langle\!\langle\sigma_1^z|\nu_1,\nu_2\rangle\!\rangle \\ \langle\!\langle\sigma_2^z|\nu_1,\nu_2\rangle\!\rangle \\ \vdots \\ \langle\!\langle\sigma_N^z|\nu_1,\nu_2\rangle\!\rangle \end{bmatrix} = \mathrm{tr}[\boldsymbol{\sigma}^{\boldsymbol{z}}\,|\nu_1\rangle\langle\nu_2|] = \frac{3}{N+1}\begin{cases}(1,-1,0,-1,\ldots,1,-1), & \text{for } N \text{even}, \\ (1,-1,0,-1,\ldots,-1,1), & \text{for } N \text{odd}.\end{cases} \tag{S16}$$

Here $\boldsymbol{\sigma}^z = (\sigma_1^z, \sigma_2^z, \ldots, \sigma_N^z)^{\mathrm{T}}$ is a vector containing the Pauli z-matrices. The magnetization eigenmode $|\epsilon\rangle^{\mathrm{s}}$ directly shows the relative phase (through the sign) and amplitude of the individual qubits' oscillations. Note that the above state corresponds to a collective mode of the many-body system.

We can verify that the synchronization conditions provided in the main text are in accordance with the necessary conditions of Ref. [S9] for quantum synchronization. The strong symmetry in our system is $A^{\mathrm{sync}} = (1/2)\,(|\nu_1\rangle\langle\nu_1| + |\nu_2\rangle\langle\nu_2|)$, where the two states spanning the decoherence-free subspace, $|\nu_{1,2}\rangle$, belong to imaginary eigenvalues of the Liouvillian. The observables of interest (the local z-polarizations) need to have non-vanishing

support on the synchronized subspace, $\mathrm{tr}\left[\sigma_j^z A\right] \neq 0$. We concretely obtain

$$\mathrm{tr}\left[\sigma_j^z A\right] = \begin{cases} 0, & \text{for } j/3 \in \mathbb{N} \\ \dfrac{2-N}{N+1}, & \text{otherwise.} \end{cases} \tag{S17}$$

We then verify permutation invariance of the synchronized z-polarizations by computing the commutator $[P_{j,k}, A]$, where $P_{j,k}$ is a permutation operator that exchanges sites j and k. We obtain

$$[P_{j,k}, A] = 0, \text{ for } \begin{cases} j,k \in \{([6m-(-1)^m - 3]/2)_{m\in\mathbb{N}}\}, \\ j,k \in \{([6m+(-1)^m - 3]/2)_{m\in\mathbb{N}}\}, \\ j = 3n\ k = 3m,\ n,m \in \mathbb{N} \end{cases} \quad \text{and } [P_{j,k}, A] \neq 0, \text{ otherwise .} \tag{S18}$$

Qubits at sites in the sequence $([6m-(-1)^m-3]/2)_{m\in\mathbb{N}} = (2,4,8,10,\ldots)$ will be identically synchronized to each other as well as qubits at sites in sequence $([6m-(-1)^m-3]/2)_{m\in\mathbb{N}} = (1,5,7,11,\ldots)$, in agreement with Eq. (S16).

The perturbative stability of quantum synchronization has been studied in Ref. [S9]. Synchronization is exponentially stable against perturbations that push the system out of the decoherence-free subspace because the noise will lead to exponential decay of any excitation normal to the invariant manifold (the asymptotic state space). On the other hand, perturbations parallel to the invariant manifold will not affect the stability of synchronization but will rather inject energy into the system, and thus might for instance change the magnitude of the oscillations.

X. Numerical simulations

The lattice model corresponding to our experiments can be described by the Hamiltonian of a one-dimensional quantum XY chain of N spins [S5]

$$H_0 = \frac{J\hbar}{2}\sum_{j=1}^{N-1}\left(\sigma_j^x\sigma_{j+1}^x + \sigma_j^y\sigma_{j+1}^y\right) + \sum_{j=1}^{N}\hbar\omega_j\sigma_j^z \tag{S19}$$

where $\sigma_j^{x,y,z}$ are the local Pauli operators acting on site j, J is nearest-neighbor coupling strength, and ω is the level spacing of the qubits. It has been shown that locally adding noise to the level spacing of a single qubit is sufficient to induce synchronization in a chain of arbitrary length N, provided a synchronization condition is satisfied. Concretely, we introduce a white noise process $\xi(t)$ with zero mean $\langle\xi(t)\rangle$ and auto-correlation $\langle\xi(t)\xi(t')\rangle = \Gamma\delta(t-t')$ via a Hermitian operator $V = \sigma_u^z$ (acting locally on site u), resulting in an exact Lindblad master equation for the evolution of the density matrix [S5]

$$\dot{\rho} = -\frac{i}{\hbar}[H_0, \rho] + \Gamma\left(V\rho V^\dagger - \frac{1}{2}\{V^\dagger V, \rho\}\right), \tag{S20}$$

where Γ denotes the strength of the noise. In order to satisfy the synchronization condition (see main text), white noise is applied to the third site $u = 3$ with $V = \sigma_3^z$. We numerically solve the master equation (S20) for ρ as a function of time from which we then compute the Pearson correlator C_{ij}, concurrence $\mathcal{C}(\rho_{15})$ and the maximally entangled mixed state fidelity $F(M, \rho_{15})$, corresponding to the theoretical curves in the main text.

[S1] Xu, Y. *et al.* High-fidelity, high-scalability two-qubit gate scheme for superconducting qubits. *Phys. Rev. Lett.* **125**, 240503 (2020).
[S2] Yan, F. *et al.* Tunable coupling scheme for implementing high-fidelity two-qubit gates. *Phys. Rev. Applied* **10**, 054062 (2018).
[S3] Tao, Z. *et al.* Interaction-induced topological pumping in a solid-state quantum system. arXiv:2303.04582.
[S4] Averin, D. V. *et al.* Suppression of dephasing by qubit motion in superconducting circuits. *Phys. Rev. Lett.* **116**, 010501 (2016).
[S5] Schmolke, F. & Lutz, E. Noise-induced quantum synchronization. *Phys. Rev. Lett.* **129**, 250601 (2022).
[S6] Gyamfi, J. A. Fundamentals of quantum mechanics in Liouville space. *Eur. J. Phys.* **41**, 063002 (2020).
[S7] Noschese, S., Pasquini, L. & Reichel, L. Tridiagonal Toeplitz matrices: properties and novel applications. *Num. Linear Algebr.* **20**, 302 (2013).

[S8] Ishizaka, S. & Hiroshima, T. Maximally entangled mixed states under nonlocal unitary operations in two qubits. *Phys. Rev. A* **62**, 022310 (2000).

[S9] Bua, B., Booker, C. & Jaksch, D. Algebraic theory of quantum synchronization and limit cycles under dissipation. *SciPost Phys.* **12**, 097 (2022).

[S10] Baumgartner, B. & Narnhofer, H. Analysis of quantum semigroups with GKS-Lindblad generators: II. General. *J. Phys. A: Math. Theor.* **41**, 395303 (2008).

[S11] Bua, B. & Prosen, T. A note on symmetry reductions of the Lindblad equation: transport in constrained open spin chains. *New J. Phys.* **14**, 073007 (2012).

[S12] Albert, V. V. & Jiang, L. Symmetries and conserved quantities in Lindblad master equations. *Phys. Rev. A* **89**, 022118 (2014).
